# Kinetic analysis of change of direction simulating a defensive action in soccer players with and without acute fatigue

**DOI:** 10.1016/j.heliyon.2024.e40213

**Published:** 2024-11-07

**Authors:** Matías de Pablo, Carol Torres, David Ulloa-Díaz, Gabriel Fábrica

**Affiliations:** a Institute of Physical Education, Universidad de la República, Montevideo, Uruguay; b Department of Sports Sciences and Physical Conditioning, Education Faculty, Universidad Católica de la Santísima Concepción, Concepción, Chile; c Department of Biophysics, Facultad de Medicina, Universidad de la República, Montevideo, Uruguay

**Keywords:** Biomechanics, Ground reaction force, Impulse, Force rate, Turning, Fatigue, Propulsion, Braking

## Abstract

The objective of this study is to analyse the kinetic effects of acute fatigue during a 45° change of direction executed with the non-dominant limb, emulating a typical defensive action during pressing in soccer. Seventeen male professional soccer players (age: 21.7 ± 5.4 years) performed a 45° change of direction before and after a fatigue protocol. Participants were instructed to execute an approach run as quickly as possible, to change direction with their non-dominant foot on a force platform, and then continue running with the aim of stopping against an opponent. Times, forces, impulses, and force rates for different subphases during support were compared. Speeds remained consistent across pre-fatigue (4.53 ± 0.55 m s-^1)^ and post-fatigue (4.58 ± 0.70 m s-^1^) trials. The results indicated a decrease in braking time (p = 0.01), impulse associated with the braking phase (p < 0.01), and impulse during body weight lifting (p = 0.01) under acute fatigue conditions. These changes suggest that, under acute fatigue, soccer players may not decelerate sufficiently in the initial direction of the run and may raise their body less in the first step after the change of direction. Maximum force values and force rates did not show significant changes; however, they were sufficiently high to suggest a potential injury risk in both conditions.

## 1 Introduction

A soccer player performs numerous changes of direction (COD) during a competitive match [[1], [2], [3], [4]] making COD useful for estimate a player's loading profile [5]. The ability to sidestep and cut quickly and effectively is critical for good offensive performance [6]. However, COD also play a crucial role in defensive pressing, and there is a notable association between pressing the ball carrier and success in a match [7]. Maneuvers involved in pressing include running forward with a COD to position oneself against an opponent who has received the ball [8]. In addition to being important for performance, this type of action is associated with musculoskeletal injuries such as anterior cruciate ligament injuries in many sports [6,9,10], and particularly in professional soccer players [8,[11], [12], [13], [14]]. Regardless of playing position, COD with angles of <90° occur more frequently than those with other angles during soccer matches [2,4].

During a COD, it is necessary to develop sufficient eccentric, isometric, and concentric muscular strength in the various subphases of support to enable rapid deceleration and subsequent reacceleration in the new intended direction [15]. These muscular contributions during the subphases of weight support, braking, and propulsion are typically associated with the vertical and anterior-posterior components of the ground reaction force (GRF) [[16], [17], [18]]. However, the medio-lateral GRF is crucial for accelerating the body's centre of mass outside the sagittal plane [3,15,18]. Although several studies have analysed GRF components during COD in soccer players, only a few have considered variables derived from the force-time curve, such as mechanical impulse [3,16,19,20], and none have examined the force rate. Mechanical impulse [19,20] and force rate [21,22] have been used as non-invasive measures of lower limb loading to explore the potential development of musculoskeletal injuries in other movements and populations. However, this approach has not yet been applied to COD in the context of defensive actions aimed at interfering with an opponent in soccer.

On the other hand, muscular contributions during a COD may vary significantly due to acute fatigue [23,24] potentially altering the kinetic variables associated with each subphase of the COD. Analysing the potential changes that acute fatigue can produce in GRF components, impulses, and force rates during COD is highly relevant. This is because it has been hypothesised that it is not the accumulated workload per se that affects lower limb mechanics and, consequently, susceptibility to injury, but rather the acute workload during relatively short and intense periods of a match [25]. An increase in GRF following fatigue can be expected, as athletes often adopt a more erect posture, and decreases in hip and knee flexion can significantly increase vertical GRF [26]. However, a classical review suggests that the effects of fatigue on GRF remain equivocal [27]. In this context, analyse a COD with 45° cutting angle is particularly interesting because it is acute enough to require substantial deceleration while still being obtuse enough to allow for a COD during a single foot contact at relatively high approach velocities [28].

Considering the arguments presented, the objective of the study is to analyse the effects of acute fatigue during a 45° COD executed with the non-dominant limb, emulating a typical defensive action during pressing in soccer, on times, forces, impulses, and force rates across various subphases of COD support. We hypothesize that acute fatigue affects kinetic variables depending on the specific subphase, support period, and direction considered.

## 2 Materials and methods

Seventeen male young professional soccer players (5 defenders, 9 midfielders and 3 forwards) from a single club volunteered to participate in the study (age: 21.7 ± 5.4 years; height: 174.3 ± 7.1 cm; body mass: 66.2 ± 8.6 kg). All participants had more than one season of experience in the first division and undertook a load of 5–7 weekly training sessions, in addition to one day of competition at the time of the study. In addition, participants were required to meet the following inclusion criteria: (1) striking the ball with the right lower extremity, (2) without any current injuries or injuries in the last 6 months, (3) unaffected by any neuromuscular problems or musculoskeletal disorders affecting the lower extremities, (4) without any respiratory or other conditions that could affect their usual physical performance, and (5) familiarized with the Rating of Perceived Exertion scale (RPE). Written informed consent was given to sign by the subjects to participate in the study. The study conforms to the Helsinki Declaration (2013) and was approved by CENUR LN of Republic University Ethics Committee (Exp. 311170-000099-21).

### 2.1 Procedures

A cross-sectional within-subject repeated measures design was used to investigate the fatigue acute effects on the kinetics during a 45° COD that simulates a common defensive play situation during pressing in soccer. We have chosen to conduct this initial study with professional soccer players from the same squad to avoid introducing potential variability factors. While we recognize that our study cannot be generalized, it is important to note that, despite its limitations, it contributes to the existing body of research. Unlike previous studies, we simulated a real game situation, and performed kinetic quantifications that considered all the subphases outlined in prior research related to support in this type of movement.

Prior to testing, the players completed a standardized 15-min warm/up divided into two parts. The first part included 7–8 min of continuous low intensity running (heart rate below 120 bpm), followed by a specific warm/up targeting the main muscle groups and movements necessary for the test execution (including three sub-maximal diagonal turning trials for familiarization). Stretching was individually managed by each player according to their sporting experience.

**Fig. 1:**
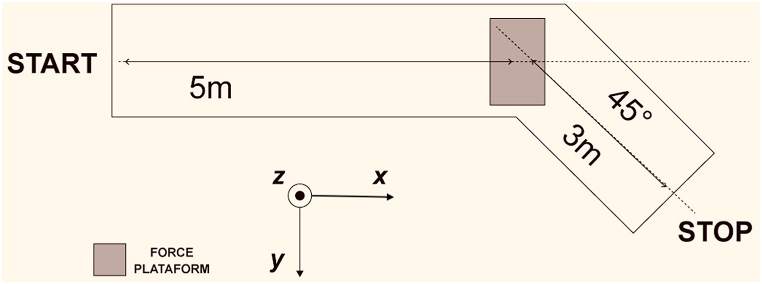
Schematic representation of the experimental setup from a top view. The x (antero-posterior), y (medio-lateral) and z (vertical) axes indicate the coordinates in the laboratory, which correspond to the directions of the platform. The z-axis is perpendicular to the plane, with its positive direction extending towards the reader.

The COD were carried out on a force platform embedded within a wooden walkway covered by artificial grass. This walkway, built specifically for the study, had an approach area of 5 m leading to the platform and an exit space after the platform, extending 3 m at a 45° angle (Fig. 1). Participants were instructed to perform the 5 m approach run as quickly as possible and change direction with their non-dominant (left) foot on the force platform (762 mm × 1016 mm ×124 mm, 1000 Hz, ATMI AccuPower-Optimized multi-axis, ATMI. Inc, USA), to continue running for an additional 3 m, with the goal of stopping at the end of the course against an opponent to simulate a defensive pressing match situation. Three consecutive trials without fatigue were performed before the subject changed to the fatigue condition. After the fatigue activity, three more trials were made. The pauses between the first trials and the fatigue protocol were set at 3 min, with no pause between the completion of the protocol and the second series of trials. Additionally, the pauses between trials were set at 1 min before fatigue and no pause during post fatigue trials [29]. Participants were instructed to maximize approach speed in both conditions and performed the trials wearing their regular synthetic field training shoes.

The acute fatigue condition was induced by performing 1 min of consecutive maximum vertical jumps [30]. This protocol was chosen because it has been successfully employed in a previous study focusing on change of direction (COD) tasks [19]; it allows for a quantifiable assessment of fatigue without invasive measures [30,31]; and finally, this procedure has been validated using physiological markers [32]. Mean mechanical power (W) was estimated and compared between the first and last 15 s of the test following the approach of Bosco et al. (1983) [30] with the expression:

$$W=\frac{g^{2}\;\text{tf}\;t}{4\;n\;\left(t-\text{tf}\right)}$$

Where g is the gravitational acceleration, tf is the sum of flight times in the analysed time period, t is the analysed time, and n is the number of jumps executed during that period.

Furthermore, to assess Rating of Perceived Exertion scale (RPE), at the end of the jumps, participants were instructed to assign a numerical value based on the CR-10 scale [33], with the following references: 0–4 indicating low exertion, 5–8 signifying moderate exertion, and 9–10 representing high exertion.

### 2.2 Data processing and variable determination

The approach speed was calculate based on the criteria outlined in Li et al. (2020) [3]. Specifically, we considered the velocity in the forward direction of the centre of mass (COM) at initial contact (heel strike). Aligns with previous research on sprints, suggesting that choosing the fastest trial is likely to yield similar outcomes to averaging the results [32], the fastest trial in each condition was used for further analysis.

A script developed in MATLAB R2018a (MathWorks, Inc.) was employed to process the acquired data and extract relevant variables. GRF in the antero-posterior direction (Fx), medio-lateral direction (Fy), and vertical direction (Fz) were smoothed using a fourth-order Butterworth low-pass digital filter with a cut-off frequency of 50 Hz [3,34]. To calculate the variables, instants of the curves graphed with the script were determined.

**Fig. 2:**
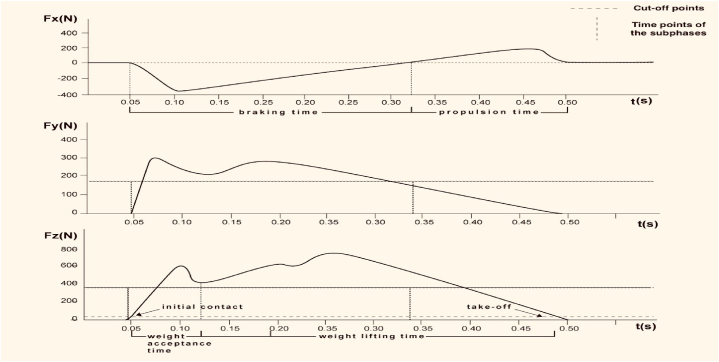
Examples of typical GRF curves observed during one trial for a single subject. The horizontal dotted lines in the upper and lower figures indicate the reference values used to identify the cut-off points that define subphases. The vertical dotted lines denote the reference values considered for defining the time periods associated with the subphases used in the calculation of the variables described in the text.

The initial contact was defined as the instance when Fz data exceeded 20 N, and take-off was defined as the instance when Fz data were below 20 N [3,35,36]. Furthermore, the support phase was divided into subphases considering two different division criteria present in the literature: braking and propulsion time based on Fx [3,18], and acceptance and lifting weight time considering Fz [15,37]. Based on these divisions, twenty variables including temporal parameters, force parameters, and impulses, considered in different previous studies [3,15,18,29,36], were analysed, their relationship with the records of the force components during the support of COD is presented in Fig. 2.

### 2.3 The variables determined in this study were

Total contact time (Tcont), determined as the difference between initial contact and take-off. Braking time (Tb), defined as the time between the start of Fx recording and the point when the magnitude of Fx becomes positive. Propulsion time (Tprop), defined as the duration from when the Fx value becomes positive until take-off. Weight acceptance time (Tacep), defined as the period from the start to the first trough in Fz. Body weight lifting time (Tlif), defined as the period to the first trough in Fz to the take-off. Minimum Fx value during braking time relative to body mass (Fxmb). Maximum Fx value during propulsion time relative to body mass (Fxmp). Maximum Fy value during contact time (Fym). Maximum Fz value during weight acceptance time relative to body mass (Fzm acc). Maximum Fz value during body weight lifting time relative to body mass (Fzm lift). Relative impulse to body mass during contact time associated with Fx (ImpTx), Fy (ImpTy), and Fz (ImpTz). Relative impulse to body mass associated with Fx during braking time (Impb) and during propulsion time (Impp). Relative impulse to body mass associated with Fz during weight acceptance (Impa) and during body weight lifting (Impl). All impulses were estimated by integrating force overtime using the trapezoidal method. Average force rates for Fx (TFx), Fy (TFy), and Fz (TFz), determined as the absolute value of the slope of the fitted line between the initial contact and the maximal absolute force values for each case.

### 2.4 Statistical analysis

Descriptive statistics were used to calculate means and standard deviations (SD) for all the variables considered in this study. The assumptions of normality and homogeneity of variance were tested using the Shapiro-Wilk test and Fisher's F-test, respectively. Differences between the pre- and post-test were analysed with the Wilcoxon test and a T test for paired samples. The effect size was calculated with the d-Cohen. The criteria for interpreting the magnitude of the effect size were as follows: >0.2 small, >0.6 moderate, and >1.2 large [38]. The statistical package JASP software (version 0.16.4) was used and in all cases, a 95 % confidence interval and a level of minor significance p < 0.05. Data will be made available on request.

## 3 Results

The approach speeds remained consistent across both conditions: 4.53 ± 0.55 m s^−1^ during pre-fatigue trials and 4.58 ± 0.70 m s^−1^ during post-fatigue trials. These values were within a range that can be considered a medium-paced running speed [39]. Acute fatigue was confirmed by a decrease in mechanical power, which decreased from 26 ± 5 to 19 ± 3 W kg^−1^ from the beginning (0–15 s) to the end (45–60 s) of the continuous jumping test. The RPE values after the fatigue protocol were always 9 or 10 for all participants. Table 1, Table 2, Table 3, Table 4 display the results of the 20 variables examined in this study, categorized by variable type (times, GRFs, impulses, and force rates).

**Table 1:** Comparison of time values for COD with and without acute fatigue.

		Before fatigue mean ± SD	After fatigue mean ± SD	*p-value*	ES
Tcont	(s)	0.24 ± 0.03	0.23 ± 0.03	0.16	0.36
Tb	(s)	0.18 ± 0.02	0.16 ± 0.02	0.01∗	−0.71
Tprop	(s)	0.06 ± 0.02	0.06 ± 0.01	0.39	−0.21
Tacep	(s)	0.04 ± 0.01	0.06 ± 0.001	0.32	0.25
Tlif	(s)	0.19 ± 0.03	0.19 ± 0.03	0.25	0.29

Tcont = total contact time; Tb = braking time; Tprop = propulsion time; Tacep = weight acceptance time; Tlif = weight lifting/time. SD = standard deviation; *p*–value = significance level; ES = Cohen's d effect size. The asterisks ∗ indicate those variables that presented significant differences when comparing between conditions.

**Table 2:** Comparison of the maximum forces for COD with and without acute fatigue.

		Before fatigue mean ± SD	After fatigue mean ± SD	*p-value*	ES
Fxmaxb	(N·kg^−1^)	4.91 ± 1.21	4.35 ± 0.82	0.14	−0.38
Fxmaxp	(N·kg^−1^)	1.20 ± 1.20	1.30 ± 0.52	0.4	0.37
Fymax	(N·kg^−1^)	4.62 ± 0.82	6.63 ± 0.88	0.88	−0.64
Fzmax accept	(N· kg^−1^)	16.29 ± 4.62	15.40 ± 2.67	0.52	0.60
Fzmax lift	(N·kg^−1^)	14.74 ± 1.97	13.99 ± 1.83	0.61	0.50

Fxmaxb = maximum braking force; Fxmaxp = maximum forward propulsion force; Fymax = maximum lateral force; Fzmax accept = maximum force during weight acceptance; Fzmax lift = maximum force during weight lifting; *p*–value = significance level; ES = Cohen's d effect size.

**Table 3:** Comparison of impulses for COD with and without acute fatigue.

		Before fatigue mean ± SD	After fatigue Mean ± SD	*p-value*	ES
ImpTx	(N·s·kg^−1^)	0.37 ± 0.13	0.29 ± 0.10	0.009∗	0.72
ImpTy	(N·s·kg^−1^)	0.72 ± 0.18	0.70 ± 0.18	0.42	0.20
ImpTz	(N·s·kg^−1^)	2.46 ± 0.40	2.26 ± 0.34	0.001∗	−4.49
Impb	(N·s·kg^−1^)	0.42 ± 0.20	0.36 ± 0.09	0.008∗	−0.73
Impp	(N·s·kg^−1^)	0.05 ± 0.04	0.06 ± 0.03	0.31	−0.26
Impa	(N·s·kg^−1^)	0.43 ± 0.13	0.40 ± 0.11	0.36	0.24
Impl	(N·s·kg^−1^)	2.03 ± 0.38	1.86 ± 0.31	0.01∗	0.68

ImpTx = total mechanical impulse in x; ImpTy = total mechanical impulse in y; ImpTz = total mechanical impulse in z; Impb = braking impulse; Impp = propulsion impulse; Impa = weight acceptance impulse; Impl = weight lifting impulse; *p*–value = significance level; ES = Cohen's d effect size. The asterisks ∗ indicate those variables that presented significant differences when comparing between conditions.

**Table 4:** Force rates for COD with and without acute fatigue.

		Before fatigue mean ± SD	After fatigue Mean ± SD	*p-value*	ES
Tfx	(N·s^−1^)	768.0 ± 349.0	786.0 ± 314.0	0.85	−0.05
Tfy	(N·s^−1^)	184.0 ± 150.6	136.7 ± 133.4	0.32	−0.25
Tfz	(N·s^−1^)	188.5 ± 51.4	201.6 ± 62.8	0.18	−0.34

Tfx = force rate in x; Tfy = force rate in y; Tfz = force rate in z; *p*–value = significance level; ES = Cohen's d effect size.

## 4 Discussion

In this study, we analysed times, maximum GRF, impulses, and force rates during support and across various subphases of support during COD at a 45° angle, simulating a defensive action during pressing in soccer. We compared these variables when the action was performed with and without acute fatigue. One noteworthy observation is the consistent approach speed across different fatigue conditions. This aspect is crucial for our study's discussion because kinetic variables during COD can vary with both angle and speed [40]. The angle was determined by the walkway's shape, but players were instructed to maximize their speed to intercept opponents in each condition. Significant differences in approach speeds between conditions could potentially obscure interpretations of the studied variables, attributing changes to the effect of fatigue on speed rather than on actions associated with different subphases. The speed registered in our study falls within the range commonly observed during changes of direction, as indicated by a recent analysis of data from Premier League matches [41].

We were able to confirm our working hypothesis, acute fatigue alters specific variables important for controlling body movement and these alterations are contingent upon the subphase and direction under consideration. Specifically, we found that the braking time was shorter in the fatigue condition, and the net impulses in the anterior-posterior and vertical axes, as well as those of the braking phase and the weight lifting phase, were significantly lower in the fatigue condition. However, none of the analysed maximum force values and force rates showed differences between conditions.

Although the approach speed developed by the soccer players in each condition can be classified as medium-paced running [41]. Total contact times fell within the range observed by other authors for athletes when running at slow linear speeds [39], and no variations after fatigue were observed in the total contact time. This is an initial suggestion of the changes in support mechanics due to the movement direction change action. Zebis et al. (2011) [42], who investigated the impact of fatigue in COD, although in handball players, also noted no significant disparities in total support times when performing COD under fatigue conditions. However, those authors noted a trend toward a slight reduction in net support time during fatigue conditions, trend that was not observed in our study. The decrease in braking phase time observed in our study suggests that during the analysed COD, similar to defensive maneuvers in pressing situations, soccer players are less able to decelerate their bodies along the anterior-posterior axis when experiencing acute fatigue compared to performing these actions without fatigue. This reduction in braking subphase times, could suggest an increased risk of ACL injury, as has been indicated in studies with vertical jumps [43]; however, we believe that it is more pertinent to discuss this with the impulse due to its mechanical meaning [44].

In regard to the analysis of maximum GRF, the obtained values were lower compared to those observed in the literature for straight runs [39] and for changes of direction by 45° performed with and without load [3], with and without fatigue in a young population not specific to any sport [29] and in female handball players [42]. However, if we consider that peak vertical GRF exceeding 3/4 of body weight can increase ACL injury risk [10,45,46]. Our results for the maximum force values obtained, especially in the vertical direction, may also indicate a risk of ACL injury regardless of whether the COD is performed with or without acute fatigue, as these values were very close to reference thresholds, particularly during the weight acceptance phase in each condition. Our findings, when comparing conditions for the maximal components of GRF, align with the study conducted by Zebis et al. (2011) [42], which reported no significant alterations in GRF components with fatigue, but do not align with the results of Cortes et al. (2014) [29]. A possible explanation for the differences with the latter may lie in the fatigue protocol used, since Cortes et al. (2014) [29] performed a fatigue protocol with treadmill runs after a VO_2_ maximum test.

Studies discussing force values in the context of fatigue in professional soccer players, have typically examined change of direction angles different from ours or analysed straight line sprints without changes of direction. In the last scenario, Wdowski et al. (2021) [47] focused on the acceleration phase of a sprint in professional soccer players. These authors observed that fatigue led to a reduction in mediolateral GRF peaks and concluded that soccer players alter their sprint mechanics to diminish lateral load and redirect force towards an increased anteroposterior and vertical direction, thereby maintaining sprint performance and reducing the risk of ankle injuries. If we extrapolate the interpretation of Wdowski et al. (2021) [47], the absence of changes in GRF values of our study suggest that professional soccer players do not necessarily need to adjust their mechanics during a 45° defensive change of direction when fatigued. However, it is important to emphasize that examining data with discrete parameters can lead to severe data reduction and loss of important information [48]. Focusing solely on maximum force values, as we have done in this study and as seen in the cited literature, may not always fully reflect the performance of an action. An analysis of the entire force-time function, as conducted in other studies [49], could offer a different perspective.

The results for the impulses showed the most interesting aspects of this work. Values obtained were close to those reported in other analyses of COD with the same angle, where other comparison factors were considered [3,16]. Significant decrease observed in the total impulse associated with anterior-posterior GRF under fatigue conditions, can be attributed to changes in the impulse during the braking phase. This, in turn, can be interpreted as linked to the observed alterations in braking time. Given that soccer players arrived at the moment of COD at the same approach speed in each condition, the decrease in braking time results in lower impulse values during fatigue condition. As mechanical impulse corresponds to the variation in the product of mass and velocity [44], a reduction of impulse implies a lesser loss of velocity of the soccer players center of mass along the anterior-posterior direction during the braking phase. This tendence to maintain speed in the original direction of approach during support in which the COD occurs, may contribute to increased shear forces at the knee level of the supporting limb. These findings align with those proposed by Zago et al. (2021) [50] in their study of soccer players performing 180° changes of direction under fatigue effects. They highlighted that a diminished contribution to braking in the penultimate step, as suggested by Dos'Santos et al. (2019) [51], could render the process less effective in dissipating impact forces, consequently overloading passive structures. Additionally, Cassiolas et al. (2023) [52] reported the highest degrees of injury risk during the braking phase, further supporting these interpretations.

On the other hand, the significant decrease in total impulse along the vertical axis was associated with changes regarding the end of support. We did not observe an increasing during the weight acceptance phase, which could involve greater effort to decelerate the downward movement of the centre of mas [53]. According to our results, the changes in vertical impulse in the COD analysed, can be attributed to the impulse reduction observed in the body weight lifting phase. Thus, in this axis, the changes occur during the push to take the first step towards the rival. This implies a reduction in the product of mass and vertical speed indicating that the athlete exhibits a lower vertical speed at the time of take-off, resulting in a shorter flight time [44]. The abbreviated flight time suggests a modification in technique at the onset of the last 3 m. In this case, unlike the discussion regarding impulse along the horizontal axis, the variation cannot be primarily attributed to changes in duration or force, as neither experienced significant change during the body weight lifting phase. Therefore, the variation in impulse is explained by the combination of minor alterations in force and time in this phase.

Considering the changes in impulse observed overall, the significance of achieving higher impulse values during the braking and vertical thrust phases, as observed in our study under non-fatigued conditions, lies in their ability to control body position during deceleration and enhance performance in the final approach towards the opponent. Following the same idea discussed in Spiteri et al. (2013) [16], the optimization of the impulse in each phase would allow athletes to decelerate earlier, applying greater impulse during the braking of the movement and to go quickly towards the opponent maximizing the vertical impulse during the lifting of the body weight.

Regarding force rates, there is currently a lack of published communication of this variable during sports movements, and to our knowledge, there is no report on these values during COD. However, our values were surprisingly high compared to those obtained in a study where running at intermediate speed was analysed to assess the effect of different footwear [21]. Taking these values as a reference, the force rates at 45° COD analysed are high in all axes, regardless of the conditions in which they are carried out. These values suggest a high risk of injury due to the fact that the degree of viscosity of the biological materials that make up the musculoskeletal system causes rigidity to increase with higher rates of force [54], thus increasing the risk of mechanical failure [54]. Finally, although the comparison of force rates suggests that fatigue does not alter this risk during this type of COD in soccer players. Laboratory based studies show that fatigue increased ligamentous laxity [55], and therefore, the same force rate could have a different consequence on biological structures such as the ACL.

Several limitations affect this study. Firstly, while our research examines a number of soccer players comparable to those included in previous studies on COD [50], and even features a larger sample size than other recent studies [3,34,36], it is important to consider a larger sample with different standards of gender, age, and experience before generalizing the applicability of the current results. Furthermore, since our analysis focused on professional soccer players from the same squad, we have a limited number of players for each position (defenders, midfielders, and forwards). As a result, we do not have a sufficient sample size to conduct a comparative analysis with playing position as a factor. It is important to analyse this in future research because, for example, midfielders are required to perform various changes in direction both before and after high-intensity efforts to meet technical and tactical requirements when in and out of possession [56,57].

On the other hand, an analysis that does not consider discrete values of force or average force rate could lead to different conclusions [49]. Furthermore, incorporating a kinematic analysis to visualize the body's orientation during the action could have broadened the discussion on whether kinetic changes are linked to different orientations during COD support. It would be interesting to investigate if other fatigue protocols yield different effects, because while continuous jumps have a solid rationale, they do not completely replicate in game situations. Additionally, testing different types of COD movements and comparing outcomes when COD is performed with the dominant versus non-dominant foot would be valuable.

## 5 Conclusions

During 45° COD, similar to defensive maneuvers performed during pressing, a significant decrease in braking time, braking impulse, and body weight lifting impulse occurs, suggesting that in conditions of acute fatigue, professional soccer players not decelerating enough in the initial direction of the run and raising the body less in the first step after the change.

During a 45° COD, similar to defensive maneuvers performed during pressing, although maximum forces and rates of force do not change significantly with the acute fatigue, it reaches values that suggest a significant risk of ACL injury in both conditions.

Although the characteristics of the analysed sample do not allow for the generalisation of these results to the entire population of soccer players, our findings suggest that professional players could benefit from training strategies designed to optimise braking and body weight-lifting impulses during COD under fatigue conditions.

## 6 Practical applications

One of the most effective methodological sequences for teaching a motor skill is: technical drills, pattern running, and then reactive agility training. Practitioners recommend that this sequence be followed without inducing fatigue, particularly for novice athletes. However, our results suggest that expert athletes may benefit from CODs under fatigue, as motor skill performance shows dynamic changes under such conditions. Although these changes do not affect peak forces and rates of force, they do reflect an increased risk of injury due to the high forces and force rates involved in COD movements. Therefore, it would be highly valuable to achieve a high level of performance in this motor skill under fatigued conditions. In this regard, it is important to consider that, beyond the duration of work, high-intensity periods determine development of acute fatigue. This should be considered when planning training activities that can lead to short-term decreases in performance of physical and technical soccer players abilities [58]. New designs for COD training should account for the fluctuations in high-intensity accelerations and decelerations changes related to player position [14], and that arise from tactical changes for example, Tierney et al. [59] report that wide midfielders perform 20 % more high-intensity decelerations in a 3-4-3 formation compared to a 4-4-2 formation.

## CRediT authorship contribution statement

**Matías de Pablo:** Writing – review & editing, Writing – original draft, Methodology, Investigation, Formal analysis, Data curation, Conceptualization. **Carol Torres:** Writing – review & editing, Writing – original draft, Visualization, Methodology, Formal analysis, Conceptualization. **David Ulloa-Díaz:** Writing – review & editing, Writing – original draft, Validation, Methodology, Formal analysis, Data curation, Conceptualization. **Gabriel Fábrica:** Writing – review & editing, Writing – original draft, Validation, Project administration, Methodology, Investigation, Formal analysis, Data curation, Conceptualization.

## Ethics statement

The study conforms to the Helsinki Declaration (2013) and was approved by CENUR LN of Republic University Ethics Committee (Exp. 311170-000099-21).

## Funding

This work was supported by the 10.13039/100008725Agencia Nacional de Investigación e Innovación, Montevideo, Uruguay [grant numbers POS_NAC_2021_1_170784].

## Declaration of competing interest

The authors declare that they have no known competing financial interests or personal relationships that could have appeared to influence the work reported in this paper.
